# Comparison of Pneumonitis Rates and Severity in Patients With Lung Cancer Treated by Immunotherapy, Radiotherapy, and Immunoradiotherapy

**DOI:** 10.7759/cureus.25665

**Published:** 2022-06-05

**Authors:** Mina Aiad, Kayla Fresco, Zarian Prenatt, Ali Tahir, Karla Ramos-Feliciano, Jill Stoltzfus, Farah Harmouch, Melissa Wilson

**Affiliations:** 1 Internal Medicine, St. Luke's University Health Network, Bethlehem, USA; 2 Internal Medicine, Penn State Health Milton S. Hershey Medical Center, Hershey, USA; 3 Internal Medicine, Lewis Katz School of Medicine at Temple University, Philadelphia, USA; 4 Hematology and Medical Oncology, St. Luke's University Health Network, Bethlehem, USA

**Keywords:** immune related adverse events, pd-1 inhibitors, pd-l1 inhibitors, ecog (eastern cooperative oncology group), cancer survival, lung cancer, radiotherapy (rt), radiation pneumonitis, drug induced pneumonitis, checkpoint inhibitor pneumonitis

## Abstract

Introduction

Radiation pneumonitis (RP) is a common dose-limiting toxicity of radiotherapy to the chest in lung cancer patients. Similarly, the revolutionary use of immune checkpoint inhibitors (ICIs) to treat lung cancer can be complicated by immune-related adverse events (irAEs), particularly checkpoint inhibitor pneumonitis (CIP). Our study aimed to assess the effect of immunotherapy, with and without radiotherapy, on pneumonitis and other outcomes.

Methods

We performed a retrospective chart review of 680 lung cancer patients treated with either radiotherapy, immunotherapy, or both at St. Luke's University Health Network to determine the incidence rates of pneumonitis. Then, a more extensive review of 346 patients was completed, 181 of whom had pneumonitis, to investigate risk factors and outcomes.

Results

All-grade pneumonitis incidence was 26.6% while more severe pneumonitis (grade 3 or higher) was 13%. Receiving programmed cell death-1 (PD-1) or ligand-1 (PD-L1) inhibitors, having squamous cell carcinoma (SCC), and having poorer performance status were independently and significantly associated with increased risk of pneumonitis, with AOR (adjusted odds ratios) of 8.32, 4.10, 2.91, and 1.71, respectively. Among those who had pneumonitis, more severe cases (grade 3 or higher) were related to immunotherapy, either alone (58.32%) or with radiation (55.7%), compared to radiation therapy alone (36.2%).

Poorer performance status (defined as a higher Eastern Cooperative Oncology Group (ECOG) score) was the only covariate we found to be significantly and independently associated with reduced odds of 18-months survival. More of the patients treated with both lung radiation and immunotherapy had progressive disease (53.8%) compared to those treated with only radiation (30.4%) or immunotherapy (36.7). Progressive disease occurred more in patients with pneumonitis grade 3 or higher (48.3%) than those with no or low-grade pneumonitis (27.2%).

Conclusion

Receiving PD-L1 and PD-1 inhibitors, either with or without radiotherapy, was associated with a higher risk of more severe pneumonitis (PD-L1 > PD-1) than radiotherapy alone. Given its high incidence and complications, more about therapy-induced pneumonitis is yet to be studied. Learning more about pneumonitis' risk factors and complications is of great clinical importance, as it may result in better treatment planning and improved outcomes. Future studies are needed to investigate the suggested association between symptomatic pneumonitis and poorer response to treatment and whether SCC increases the risk of higher-grade pneumonitis.

## Introduction

Pneumonitis is a general term used to describe (non-infectious) inflammation of lung tissue. With developing and approving more promising therapies for lung cancers come more challenging side effects and toxicities, including pneumonitis. Both chest radiation and immunotherapy (ICIs) were significantly associated with pneumonitis, with a higher risk than that of chemotherapy [1]. The reported incidence of pneumonitis seems to vary widely based on the treatment regimen, including the immunotherapy used, radiation dose, and frequency [2]. Previous studies concluded that the actual incidence of pneumonitis in the real world is still unknown and could be higher than that reported in clinical trials [3-4]. Aside from the therapy(ies) used, various patient- and disease-related risk factors were identified [5].

Pneumonitis is clinically significant because it is a dose-limiting toxicity of radiotherapy, and it also can delay or even terminate immunotherapy [6]. The severity of pneumonitis was graded on a scale of 1 to 5 according to the National Cancer Institute CTCAE (Common Terminology Criteria for Adverse Events) version 5.0. Grades 3 and 4 represent severe and life-threatening symptoms, respectively, and pneumonitis could be fatal sometimes (Grade 5). Prior studies suggested that pneumonitis is the most frequent cause of lung cancer therapy-related mortality, with an early mortality rate of 36% [7-8].

Despite its clinical importance and seriousness, comprehensive knowledge of pneumonitis was not achieved until typing this report [9]. The focus of this retrospective study was to assess the effect of immunotherapy (with or without radiation) on pneumonitis incidence and severity. We aimed to evaluate different patients, diseases, and therapy-related variables, including pneumonitis, and their association with lung cancer treatment outcomes.

## Materials and methods

Patients with a diagnosis of lung cancer between July 2015 and July 2021 at St. Luke's University Health Network were identified on an IRB-approved protocol (IRB 00002757 issued approval SLIR 2021-58). We excluded patients who were not treated with immunotherapy or lung radiation, as well as those who lacked enough data about their treatment or follow-up after their diagnoses of lung cancer. We conducted an initial chart review focused on determining pneumonitis incidence and grades of severity within six months after receiving either immunotherapy or radiation. Pneumonitis cases were identified according to the assessment of the treating providers, along with reported symptoms, radiological findings, and ruling out other differentials.

Given that we had a relatively larger sample size of those who did not have pneumonitis, we randomly selected a sample out of them (using systemic sampling) to serve as a control group for the others with pneumonitis. We then conducted a more extensive retrospective chart review to investigate variables previously identified as risk factors for pneumonitis and evaluate clinical outcomes.

Patient-related factors included age and performance status at the time of diagnosis, gender, race, smoking history and status, pre-existing chronic obstructive pulmonary disease (COPD), emphysema, and interstitial lung disease (ILD). Cancer-related factors included lung cancer type and stage at diagnosis. Therapy-related factors included the immunotherapy used, total radiation dose in gray (Gy) and fractions, and the type of therapy received six months before pneumonitis onset.

Primary outcomes included the duration of survival in months after both diagnoses of lung cancer and pneumonitis and the disease response based on Revised Response Evaluation Criteria in Solid Tumors (RECIST) criteria version 1.1. We used RECIST 1.1, not the modified immune-based RECIST (iRECIST) because we reviewed charts predated iRECIST publication in 2017. Thus, we avoided reclassifying and reevaluating lung cancer response to therapy using different criteria. All charts were reviewed at least twice by two different abstractors, matched, and confirmed to increase data collection's inter-rater reliability and objectivity.

For our primary outcome of pneumonitis incidence and severity, we created a binary dependent variable (Grades 0-2 as "no" versus 3-5 as "yes") to conduct multivariable direct logistic regression with sufficient subgroup samples. Our goal was to determine the independent contribution of treatment type after adjusting for relevant demographic and clinical variables. Next, for just patients with pneumonitis, we conducted a chi-square test to evaluate the association between the type of treatment received within six months of pneumonitis onset (radiation, immunotherapy, or both) and pneumonitis severity (coded 1 to 4, with four representing Grades 4 and 5 combined). Finally, we presented descriptive outcomes for the association between seven specific types of immunotherapies (including PD-1/PD-L1 and CTLA-4 (cytotoxic T-lymphocyte associated protein) inhibitors) and grade of pneumonitis severity, given the limited subgroup samples in patients who received immunotherapy.

Before logistic regression modeling, we conducted separate bivariate analyses comparing each potential covariate and our outcome to determine which variables were best suited to multivariate modeling at p < 0.10. We chose this more conservative p-value to help ensure adequate subgroup sample sizes for modeling, especially given our limited number of pneumonitis grade ≥ 3 events (n = 89). In addition to treatment type, our potential covariates were selected based on previous research and clinical considerations. They included the following: age at the time of lung cancer diagnosis, gender, race, smoking within five years of lung cancer diagnosis, smoking pack-years history, pre-existing COPD, pre-existing emphysema, performance status at the time of diagnosis, type of lung cancer, stage of lung cancer, lung radiation dose (Gy) and fractions within six months of pneumonitis, and total lifetime lung radiation (Gy) and fractions. Performance status was determined according to the Eastern Cooperative Oncology Group (ECOG) score.

For both age and ECOG score as continuous covariates, linearity in the logit was satisfied based on examining the scatterplot of predicted probabilities and the difference between observed and predicted model probabilities. Additionally, we assessed the presence of outliers and influential data points. There were only 11/346 outliers (3.2%) and 0/346 influential data points based on examination of the normalized residuals, Cook's D, and leverage statistics. Therefore, we retained all patients in this model.

We reported the omnibus chi-square statistic and the Hosmer-Lemeshow goodness-of-fit statistic to ascertain model fit. We present adjusted odds ratios (AOR) for each covariate and 95% confidence intervals (CIs).

As a secondary outcome, we assessed 18-months survival after lung cancer diagnosis using a direct multivariable logistic regression model with the previous patient demographic and clinical variables significant at p < 0.10 following bivariate analysis. For the age and ECOG score as continuous covariates, linearity in the logit was satisfied, with only 7/346 outliers (2.0%) and 0/346 influential data points. Therefore, we retained all patients in this model.

As other secondary outcomes, we determined differences in RECIST criteria (progressive disease versus others) based on the type of treatment received within six months of pneumonitis onset (radiation, immunotherapy, or both) and presence of pneumonitis (grade ≥ 3) using separate chi-square tests.

We used SPSS version 28 (Armonk, NY: IBM Corp.) to analyze our data, with p < 0.05 denoting statistical significance for all outcomes and no adjustment for the multiple comparisons. Additionally, study data were collected and managed using REDCap (research electronic data capture) electronic data capture tools hosted at St. Luke's University Health Network (SLUHN).

## Results

**Figure 1:**
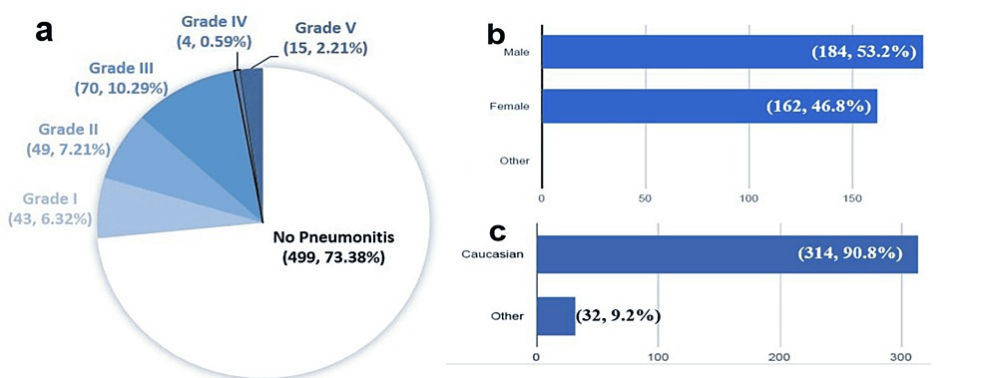
(a) Pneumonitis incidence and grades of severity (N=680; n, %). (b) Gender (N=346; n, %). (c) Race (N=346; n, %) Modified from our REDCap data analysis report

Six-hundred eighty (680) lung cancer patients with the above criteria (lung cancers diagnosed between July 2015 and July 2021 who were treated with radiation therapy, immunotherapy, or both at SLUHN) were identified. All-grade pneumonitis occurred in 181 patients (26.6%) while Grade 3 or higher occurred in only 89 (13.1%) of them (Figure 1a). We performed a complete chart review of 346 patients, including the 181 patients with pneumonitis and 165 randomly selected patients who did not have pneumonitis (as a control group). The mean age was 69.5 years ± 9.9; 162 females (46.8%), 184 males (53.2%); 314 Caucasian (90.8%), and 32 non-Caucasian (9.2%) (Figures 1b-1c).

Table 1 presents bivariate comparisons of patients' demographic and clinical variables for pneumonitis as a binary outcome. Three variables had p-values of statistically high significance (≤ 0.001): type of immunotherapy, total lifetime lung radiation (Gy), and stage of lung cancer at diagnosis. In addition, the following covariates were included in the multivariable regression model (p < 0.10): age, gender, type of lung cancer, and ECOG score.

**Table 1:** Bivariate comparisons of demographic and clinical variables for pneumonitis (a) p-value: probability-value; Based on separate chi-square tests for categorical variables, Student’s t-test for age as a normally distributed continuous variable, and Mann Whitney rank-sum test for ECOG score as an ordinal variable. (b) PD-1: programmed cell death-1; (c) PD-L1: programmed death-ligand 1; (d) COPD: chronic obstructive pulmonary disease; (e) ECOG: Eastern Cooperative Oncology Group; (f) Gy: Gray, a unit of ionizing radiation dose in the International System of Units (SI)

	Pneumonitis: grade ≥3 (n = 89)	No pneumonitis: grade 0-2 (n = 257)	p-value ^(a)^
Type of Immunotherapy (n, %)	Neither (14, 8.8%)	Neither (146, 91.3%)	< 0.001
	Anti-PD1 ^(b)^ (53, 46.1%)	Anti-PD1 (62, 53.9%)	
	Anti-PDL1 ^(c)^ (22, 31.0%)	Anti-PDL1 (49, 69.0%)	
Age in years (mean ± standard deviation)	(68.1 ± 10.0)	(70.0 ± 9.8)	0.07
Gender (n, %)	Female (33, 20.4%)	Female (129, 79.6%)	0.03
	Male (56, 30.4%)	Male (128, 69.6%)	
Race (n, %)	Caucasian (84, 26.8%)	Caucasian (230, 73.2%)	0.17
	Non-Caucasian (5, 15.6%)	Non-Caucasian (27, 84.4%)	
Smoking within 5 Years of Lung Cancer Diagnosis (n, %)	Yes (52, 27.1%)	Yes (140, 72.9%)	0.52
	No (37, 24.0%)	No (117, 76.0%)	
Smoking Pack-Years (n, %)	0-10 pack-years (7, 16.7%)	0-10 pack-years (35, 83.3%)	0.24
	10-40 pack-years (32, 24.4%)	10-40 pack-years (99, 75.6%)	
	> 40 pack years (50, 28.9%)	> 40 pack years (123, 71.1%)	
Pre-existing COPD ^(d) ^(n, %)	None, mild, or unknown (42, 26.1%)	None, mild, or unknown (119, 73.9%)	0.89
	Moderate to very severe (47, 25.4%)	Moderate to very severe (138, 74.6%)	
Pre-existing Emphysema (n, %)	None, mild, or unknown (65, 25.1%)	None, mild, or unknown (194, 74.9%)	0.65
	Moderate to very severe (24, 27.6%)	Moderate to very severe (63, 72.4%)	
ECOG ^(e)^ Score (Median, range)	1 (0 – 3)	1 (0 – 4)	0.01
Type of Lung Cancer (n, %)	Adenocarcinoma (39, 25.8%)	Adenocarcinoma (112, 74.2%)	0.02
	Squamous cell (36, 34.0%)	Squamous cell (70, 66.0%)	
	Small cell (8, 23.5%)	Small cell (26, 76.5%)	
	Other (6, 11.1%)	Other (48, 88.9%)	
Stage of Lung Cancer (n, %)	Stage I, II, or limited (22, 15.5%)	Stage I, II, or limited (120, 84.5%)	< .001
	Stage III, IV, or extensive (67, 32.8%)	Stage III, IV, or extensive (137, 67.2%)	
Lung Radiation (Gy)^ (f)^ Within 6 Months of Pneumonitis (n, %)	None (34, 55.7%)	None (27, 44.3%)	0.13
	1-30 Gy (8, 57.1%)	1-30 Gy (6, 42.9%)	
	31-60 Gy (41, 41.8%)	31-60 Gy (57, 58.2%)	
	> 60 Gy (6, 75.0%)	> 60 Gy (2, 25.0%)	
Lung Radiation Fraction Within 6 Months of Pneumonitis (n, %)	None (34, 55.7%)	None (27, 44.3%)	0.31
	< 15 (15, 39.5%)	< 15 (23, 60.5%)	
	15-30 (33, 46.5%)	15-30 (38, 53.5%)	
	> 30 (7, 63.6%)	> 30 (4, 36.4%)	
Total Lifetime Lung Radiation Gy (n, %)	< 30: 25 (42.4%)	< 30: 34 (57.6%)	0.001
	> 30 (64, 22.3%)	> 30 (223, 77.7%)	
Total Lifetime Lung Radiation Fraction (n, %)	< 15 (41, 22.4%)	< 15 (142, 77.6%)	0.14
	> 15 (48, 29.4%)	> 15 (115, 70.6%)	

Table 2 presents the multivariable logistic regression results. As indicated in the table footnote, the model had adequate goodness of fit based on the omnibus chi-square and Hosmer-Lemeshow p-values. The overall correct classification rate was 75.6% based on the included covariates. As presented in Table 2, the following four factors were independently and significantly associated with increased risk of pneumonitis: anti-PD1 (AOR = 8.32), anti-PDL1 (AOR = 4.10), a higher ECOG score (AOR = 1.71), and squamous cell carcinoma (AOR = 2.91).

**Table 2:** Multivariable logistic regression for pneumonitis (a) AOR: adjusted odds ratio; CI: confidence interval. (b) p-value: probability-value; Omnibus chi-square p-value < 0.001; Hosmer-Lemeshow goodness-of-fit p-value = 0.28. (c) PD-1: programmed cell death-1; (d) PD-L1: programmed death-ligand 1; (e) ECOG: Eastern Cooperative Oncology Group; (f) Gy: Gray, a unit of ionizing radiation dose in the International System of Units (SI)

Covariates	AOR (95% CI) ^(a)^	p-value ^(b)^
Type of Immunotherapy (Reference = none)	Anti-PD1 ^(c)^: 8.32 (3.86 – 17.93)	< 0.001
	Anti-PDL1 ^(d)^: 4.10 (1.76 – 9.55)	0.001
Age	0.99 (0.96 – 1.02)	0.51
Gender (Reference = female)	1.35 (0.76 – 2.41)	0.30
ECOG ^(e)^ score	1.71 (1.14 – 2.56)	0.01
Type of Lung Cancer (Reference = other types)	Adenocarcinoma: 1.47 (0.53 – 1.40)	0.46
	Squamous cell carcinoma: 2.91 (1.02 – 8.29)	0.05
	Small cell carcinoma: 2.07 (0.56 – 7.71)	0.28
Stage of Lung Cancer (Reference = Stage I, II, or limited)	1.10 (.55 – 2.17)	0.79
Total Lifetime Lung Radiation Gy ^(f)^ (Reference ≤ 30)	0.64 (0.32 – 1.29)	0.21

Tables 3-4 present the subgroup analyses for pneumonitis patients. In Table 3, the association between the type of treatment received within six months of pneumonitis and its grade of severity was statistically significant (p = .01). Only 36.2% of radiation-only patients had Grade 3 or higher pneumonitis, compared to 58.3% and 55.7% of patients who received immunotherapy-only and immunotherapy with radiation, respectively. Table 4 gives the descriptive outcomes for the type of immunotherapy and grade of pneumonitis severity. More patients who received PD-1 inhibitors (59.5%) had Grade 3 or higher pneumonitis than PD-L1 inhibitors (43.1%).

**Table 3:** Association between recent treatment (within six months) and pneumonitis grade

	Statistics	Grade 1	Grade 2	Grade 3	Grades 4 or 5	Total
Radiation	Count	24	20	17	8	69
	% Within Treatment Type	34.8%	29.0%	24.6%	11.6%	100.0%
Immunotherapy	Count	6	19	30	5	60
	% Within Treatment Type	10.0%	31.7%	50.0%	8.3%	100.0%
Both	Count	13	10	23	6	52
	% Within Treatment Type	25.0%	19.2%	44.2%	11.5%	100.0%
Total	Count	43	49	70	19	181
	% Within Treatment Type	23.8%	27.1%	38.7%	10.5%	100.0%

**Table 4:** Association between the type of immunotherapy and pneumonitis grade (a) PD-1: programmed cell death-1; (b) PD-L1: programmed death-ligand 1; (c) both “3. Anti-Cytotoxic T-Lymphocyte Associated Protein-4 (CTLA-4): Ipilimumab and Atezolizumab”, and “7. Anti-cluster of differentiation 20 (CD20): Rituximab” are not shown because each had no patients; (d) EGFR: epidermal growth factor receptor; (e) VEGF: vascular endothelial growth factor. (f) RANKL: Receptor Activator of Nuclear Factor-kappa B

	Statistics	Grade 1	Grade 2	Grade 3	Grades 4 or 5	Total
1. Anti-PD1 ^(a)^ (Nivolumab and Pembrolizumab)	Count	14	22	44	9	89
	% Within Treatment Type	15.7%	24.7%	49.4%	10.1%	100.0%
2. Anti-PDL1 ^(b)^ (Durvalumab and Atezolizumab)	Count	14	15	17	5	51
	% Within Treatment Type	27.5%	29.4%	33.3%	9.8%	100.0%
4^(c)^. Anti-EGFR ^(d) ^(Cetuximab)	Count	0	0	1	0	1
	% Within Treatment Type	0.0%	0.0%	100.0%	0.0%	100.0%
5. Anti-VEGF ^(e) ^(Bevacizumab and Ramucirumab)	Count	1	1	1	0	3
	% Within Treatment Type	33.3%	33.3%	33.3%	0.0%	100.0%
6. Anti-RANKL ^(f)^ (Denosumab)	Count	0	1	0	0	1
	% Within Treatment Type	0.0%	100.0%	0.0%	0.0%	100.0%
Total	Count	29	39	63	14	145
	% Within Treatment Type	20.0%	26.9%	43.4%	9.7%	100.0%

Table 5 presents bivariate comparisons for survival at 18 months after a lung cancer diagnosis. Both ECOG score and cancer stage at diagnosis had highly significant p-values, <0.001 and 0.005, respectively. Additional three covariates were included in the multivariable regression model: pre-existing COPD, cancer type, and total lifetime lung radiation (p-values of 0.02, 0.08, and 0.07, respectively). While the type of immunotherapy suggested a relationship with pneumonitis in the previous analysis (Table 1), it had no significant relationship with survival (p = 0.24).

**Table 5:** Bivariate comparisons of demographic and clinical variables for 18-month survival after a lung cancer diagnosis (a) Ten patients were followed for less than 18 months, as they were alive at the time of data collection with a survival duration between eight and 15 months. (b) Based on separate chi-square tests for categorical variables, student’s t-test for age as a normally distributed continuous variable, and Mann-Whitney rank sums test for ECOG score as an ordinal variable. (c) PD-1: programmed cell death-1; (d) PD-L1: programmed death ligand-1; (e) COPD: chronic obstructive lung disease; (f) ECOG: Eastern Cooperative Oncology Group; (g) Gy: Gray, a unit of ionizing radiation dose in the International System of Units (SI)

	Alive at 18 Months (n = 217)	Deceased at 18 Months^(a)^ (n = 128)	p-value ^(b)^
Type of Immunotherapy (n, %)	Neither (107, 67.3%)	Neither (52, 32.7%)	0.24
	Anti-PD1 ^(c)^ (70, 60.9%)	Anti-PD1 (45, 39.1%)	
	Anti-PDL1 ^(d)^ (40, 56.3%)	Anti-PDL1 (31, 43.7%)	
Age, years (mean ± standard deviation)	(69.0 ± 9.6)	(70.3 ± 10.3)	0.24
Gender (n, %)	Female (103, 64.0%)	Female (58, 36.0%)	0.70
	Male (114, 62.0%)	Male (70, 38.0%)	
Race (n, %)	Caucasian (200, 63.9%)	Caucasian (113, 36.1%)	0.23
	Non-Caucasian (17, 53.1%)	Non-Caucasian (15, 46.9%)	
Smoking within 5 Years of Cancer Diagnosis (n, %)	Yes (114, 59.4%)	Yes (78, 40.6%)	0.13
	No (103, 67.3%)	No (50, 32.7%)	
Smoking Pack-Years (n, %)	0-10 pack-years (26, 63.4%)	0-10 pack years (15, 36.6%)	0.67
	10-40 pack-years (86, 65.6%)	10-40 pack years (45, 34.4%)	
	> 40 pack years (105, 60.7%)	> 40 pack years (68, 39.3%)	
Pre-existing COPD ^(e)^ (n, %)	None, mild, or unknown (90, 56.3%)	None, mild, or unknown (70, 43.7%)	0.02
	Moderate to very severe (127, 68.6%)	Moderate to very severe (58, 31.4%)	
Pre-existing Emphysema (n, %)	None, mild, or unknown: 163 (62.9%)	None, mild, or unknown (96, 37.1%)	0.98
	Moderate to severe (54, 62.8%)	Moderate to severe (32, 37.2%)	
ECOG ^(f)^ Score (Median, range)	(1, 0 – 3)	(1, 0 – 4)	< 0.001
Type of Lung Cancer (n, %)	Adenocarcinoma (88, 58.7%)	Adenocarcinoma (62, 41.3%)	0.08
	Squamous cell 69 (65.1%)	Squamous cell (62, 41.3%)	
	Small cell (18, 52.9%)	Small cell (16, 47.1%)	
	Other: (41, 75.9%)	Other (13, 24.1%)	
Stage of Lung Cancer (n, %)	Stage I, II, or limited 101 (71.6%)	Stage I, II, or limited (40, 28.4%)	0.005
	Stage III, IV, or extensive (116, 56.9%)	Stage III, IV, or extensive (88, 43.1%)	
Lung Radiation Gy ^(g)^ within 6 Months of Pneumonitis (n, %)	None (42, 68.9%)	None (19, 31.1%)	0.45
	1-30 Gy (9,64.3%)	1-30 Gy (5, 35.7%)	
	31-60 Gy (56, 57.1%)	31-60 Gy (42, 42.9%)	
	> 60 Gy (4, 50.0%)	> 60 Gy (4, 50.0%)	
Lung Radiation Fraction within 6 Months of Pneumonitis (n, %)	None (42, 68.9%)	None (19, 31.1%)	0.12
	< 15 (19, 50.0%)	< 15 (19, 50.0%)	
	15-30 (41, 57.7%)	15-30 (30, 42.3%)	
	> 30 (9, 81.8%)	> 30 (2, 18.2%)	
Total Lifetime Lung Radiation Gy (n, %)	< 30 (31, 52.5%)	< 30 (34, 57.6%)	0.07
	> 30 (186, 65.0%)	> 30 (100, 35.0%)	
Total Lifetime Lung Radiation Fraction (n, %)	< 15 (117, 64.3%)	< 15 (65, 35.7%)	0.57
	> 15 (100, 61.3%)	> 15 (63, 38.7%)	

Table 6 presents the multivariable logistic regression results. As indicated in the table footnote, the model had adequate goodness of fit based on the omnibus chi-square and Hosmer-Lemeshow p-values. However, the overall correct classification rate was only 67.7% based on the included covariates. As presented in Table 6, a higher ECOG score was the only covariate independently and significantly associated with reduced odds of survival at 18 months (AOR = 0.52). Although both small cell lung cancer (SCLC; AOR = 0.39) and advanced lung cancer stage (AOR = 0.60) were also associated with reduced odds of survival, their level of significance was borderline (p = 0.06 for both).

**Table 6:** Multivariable logistic regression for 18-month survival after a lung cancer diagnosis (a) AOR: adjusted odds ratio; CI: confidence interval. (b) p-value: probability-value; Omnibus chi-square p-value <0.001; Hosmer-Lemeshow goodness-of-fit p-value = 0.37

Covariates	AOR (95% CI) ^(a)^	p-value ^(b)^
Pre-existing COPD ^(a)^	1.50 (0.91 – 2.46)	0.11
ECOG ^(b)^ score	0.52 (0.37 - 0.74)	< 0.001
Type of Lung Cancer (reference = other types)	Adenocarcinoma 0.55 (0.27 – 1.14)	0.11
	Squamous cell 0.68 (0.31 – 1.47)	0.33
	Small cell 0.39 (0.15 – 1.02)	0.06
Stage of Lung Cancer (reference = stages I, II, or limited)	0.60 (0.36 – 1.02)	0.06
Total Lifetime Lung Radiation Gy ^(c)^ (reference = none)	1.07 (0.57 – 2.01)	0.83

Tables 7-8 present our additional secondary outcomes analyses. In Table 7, there was a significant association between RECIST score categories and the type of therapy received within six months of pneumonitis. Patients treated with both radiation and immunotherapy had more progressive disease (53.8%) compared to those who received radiation (30.4%) or immunotherapy (36.7%) alone. In Table 8, there was a significant association between RECIST score categories and pneumonitis. More progressive disease occurred in patients with pneumonitis grade 3 or above (48.3% versus 27.2% for Grades 0-2).

**Table 7:** RECIST criteria based on the type of treatment within six months of pneumonitis diagnosis (a) RECIST: Response Evaluation Criteria in Solid Tumors. (b) p-value: probability-value; based on the chi-square test

RECIST ^(a)^ Criteria			
	Progressive Disease (PD) (n = 71)	All Other Scores (n = 110)	P-value ^(b)^
Radiation (n, %)	(21, 30.4%)	(48, 69.6%)	0.03
Immunotherapy (n, %)	(22, 36.7%)	(38, 63.3%)	
Both (n, %)	(28, 53.8%)	(24, 46.2%)	

**Table 8:** RECIST criteria based on pneumonitis diagnosis. (a) RECIST: Response Evaluation Criteria in Solid Tumors. (b) p-value: probability-value; based on the chi-square test

RECIST ^(a)^ Criteria			
	Progressive Disease (PD) (n = 113)	All Other Scores (n = 233)	P-value ^(b)^
Pneumonitis Grade 0-2	70 (27.2%)	187 (72.8%)	< 0.001
Pneumonitis Grade ≥ 3	43 (48.3%)	46 (51.7%)	

## Discussion

**Figure 2:**
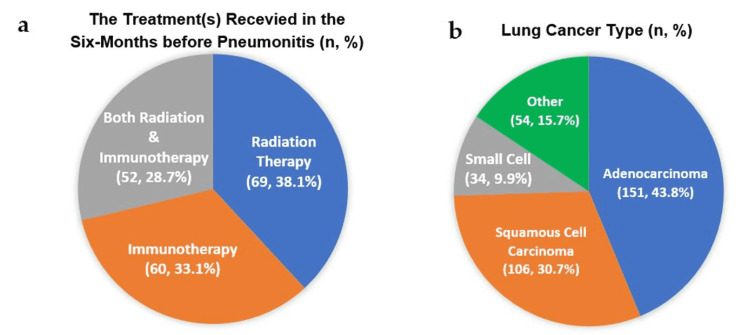
(a) Recent treatment (within six months) before pneumonitis onset. (b) Lung cancer type Modified from our REDCap data analysis report REDCap: research electronic data capture

The two most common cancer therapies that induce pneumonitis are chest radiation and immunotherapy, with a higher risk than chemotherapy [1]. Only a few studies addressed chemotherapy-related pneumonitis [8]. We decided to include radiation and immunotherapy and compare the incidence and severity of pneumonitis among three treatment groups: radiation-only, immunotherapy-only, and combined radiation/immunotherapy (Figure 2a). Most previous studies focused on each therapy apart, either radiation pneumonitis (RP) or immune-related pneumonitis (IRP). To the best of our knowledge, only one previous study, by Voong et al., addressed the relationship between prior radiotherapy and subsequent checkpoint inhibitor pneumonitis (CIP) in advanced non-small-cell lung cancer (NSCLC) [3]. Unlike Voong's study, the inclusion criteria for our research were neither limited to receiving radiotherapy before PD-1/PD-L1 inhibitors nor advanced NSCLC (Figure 2b).

The incidence of pneumonitis in our study was higher than that reported in Voong's study, which was reported to be higher than rates in published clinical trials. Our all-grade pneumonitis incidence was 26.6% (181, N=680) while the incidence of pneumonitis Grade ≥3 was 13% (89, N=680) as compared to 19% and 11% (36 and 21, N=188) in Voong’s study. Our RP incidence was much lower and not consistent with the reported 40% in the Palma et al. study [10]. However, the 40% referred to the estimated average range of 13% to 37% based on findings of two other studies; the first had a protocol of high radiation dose (66 Gy) and the second used a calculation that "overestimated the actual observed rated for RP" [11].

Regarding the risk factors, we identified four independent variables that were significantly associated with an increased risk of pneumonitis Grade 3 or higher (Table 9). PD-1 or PD-L1 inhibitors had adjusted odds ratios (AOR) of 8.32 and 4.10, respectively. Again, a subgroup analysis of the pneumonitis cases revealed that more patients (59.5%) of PD-1 inhibitors had Grade 3 or higher pneumonitis than PD-L1 inhibitors (43.1%). In other words, receiving PD-1 inhibitors was associated with a higher incidence and severity of pneumonitis than receiving PD-L1 inhibitors. These findings are consistent with prior studies. A large retrospective data review of 5,038 NSCLC patients who received PD-1 (n=3,232) and PD-L1 (n=1,806) inhibitors found significantly higher incidence of pneumonitis with PD-1 (3.6%) than with PD-L1 (1.3%) inhibitors [12].

**Table 9:** Variables found to be independently and significantly associated with Grade three or higher pneumonitis Adapted from Table 2 (a) p-value: probability-value; (b) PD-1: programmed cell death-1; (c) PD-L1: programmed death ligand-1

Variable	Adjusted Odds Ratio	p-value^(a)^
PD-1 ^(b)^ Inhibitor	8.32	<0.001
PD-L1 ^(c)^ Inhibitor	4.10	0.001
Squamous Cell Lung Carcinoma	2.91	0.05
ECOG Score	1.71	0.01

The third risk factor associated with pneumonitis grade or higher was squamous cell carcinoma (SCC) of the lung, with AOR 2.91, compared to other types of lung cancer, including SCLC and NSCLC. We found only one prior study, a retrospective review of 205 patients, suggesting that tumor histological type, namely, SCC, is a potential risk factor for CIP pneumonitis [4]. While prior studies explained the roles of genetic susceptibility and cytokine upregulations in developing pneumonitis [2,5], there was no explanation for the relationship between different cancer histological types and the pathogenesis of therapy-induced pneumonitis.

A higher ECOG score (poorer PS) at lung cancer diagnosis was also significantly and independently associated with Grade 3 or higher pneumonitis, with AOR1 1.71. To our knowledge, this is the first study to report a higher ECOG score as a risk factor for developing therapy-induced pneumonitis. If confirmed clinically significant, it may influence the treatment planning for those deemed high-risk for pneumonitis. Not only was poorer performance status associated with more severe pneumonitis, but pneumonitis, in turn, resulted in worse performance status among its survivors [8]. Those with poorer performance could be at higher risk of therapy-induced pneumonitis, and even if they survive, their PS is likely to get even poorer.

Our study also examined the chronological relationship between receiving specific types of treatment and developing pneumonitis to assess the inciting cause. A subgroup analysis for pneumonitis patients revealed that more than half of those who received immunotherapy (either alone: 58.3% or with lung radiotherapy: 55.7%) had a high-grade pneumonitis (Grade 3 or higher), compared to only 36.2% of those treated with radiotherapy. As noted earlier, most of the previous studies have evaluated the CIP and RP separately. The study by Voong et al. addressed both therapies as risk factors. Still, it focused on prior radiation therapy and its effect on pneumonitis incidence among patients with advanced NSCLC receiving immunotherapy [3]. To our knowledge, our study is the first to compare the prevalence of high-grade pneumonitis in the three treatment groups: immunotherapy, radiotherapy, and both.

As a secondary outcome of our study, we found that a higher ECOG score (poor PS) at diagnosis of lung cancer was the only covariate independently and significantly associated with reduced odds of survival at 18 months (AOR = 0.52). A prior study that analyzed 1,655 outpatients with advanced cancers found that using PS (ECOG score) alone as a prognostic tool for survival is as good as using more complex models like the palliative performance scale (PPS) and Karnofsky performance status (KPS) [13]. Therefore, we highlight the importance of using the ECOG score and incorporating it into shared decision-making and treatment planning for more realistic goals and expectations. Other variables that showed a trend with reduced odds of survival at 18 months of diagnosis but did not reach statistical significance were small cell lung cancer and advanced stage (II or III NSCLC or extensive SCLC). Despite our dataset's lack of statistical significance, they are well-established negative prognostic factors for survival [14].

A finding that could be of interest, although inconsistent with several previous studies, is that a higher percentage (53.8%) of our lung cancer patients who received both lung radiation and immunotherapy had progressive disease compared to those who received either radiation (30.4%) or immunotherapy (36.7%) alone [15]. This could be in part due to our limited sample size. A phase-II randomized trial (N=76) of pembrolizumab (PD-1 inhibitor) after stereotactic body radiotherapy (SBRT) (n=36) vs. pembrolizumab alone (n=40) in advanced NSCLC concluded that radioimmunotherapy was associated with improvement in overall response rate at 12 weeks [16]. Similarly, a secondary analysis of the KEYNOTE-001 phase 1 trial suggests a more prolonged progression-free survival and overall survival with radiotherapy subsequent with pembrolizumab compared to pembrolizumab alone [17]. Ongoing randomized trials explore the combined radiotherapy and immunotherapy versus immunotherapy alone in lung cancer patients [18]. However, clinical trials are not real-life settings, and their participants may not represent the overall population. Further studies are yet to analyze further the potential synergetic effect and risks of combining radiotherapy with immunotherapy.

Previous studies found pneumonitis associated with poor prognosis, worse performance status, and increased mortality [8,19]. Our study's relevant but newly reported finding is a significant association between high-grade pneumonitis and poor response to therapy. More of those with Grade 3 or higher pneumonitis (48.3%) had progressive disease (as defined by RECIST criteria) compared to the others who had no or low-grade (I-II) pneumonitis (27.2%). If proven clinically significant, this association needs to be studied further, especially at histological and biochemical levels, in the context of pneumonitis pathogenesis. Whether reversing the inflammatory and fibrotic changes of pneumonitis would result in a better response to cancer therapies is of clinical importance, and further studies are needed to explore it.

Our study was limited to a single health network population, with slightly over 90.8% of subjects identified as caucasian. Thus, it might underestimate other groups, especially non-caucasian. The sample size could be larger, especially with our limited number of pneumonitis cases identified. Immunotherapies subgroups different from PD-L1 and PD-1 inhibitors did not have enough patients to be included in the analysis. Additionally, being a retrospective study has its own intrinsic limitations such as dependence on previous data and documentation. For example, a few patients had no documented COPD history and therefore were added to the "none, mild or unknown group." Despite addressing the type of immunotherapy and whether chemotherapy was used, we did not include the immunotherapy dose, adherence, or the type of chemotherapy used, partially due to the nature of our data collection and insufficient subgroup sizes. Further studies would be helpful in investigating and identifying differences in these subgroups.

## Conclusions

Pneumonitis is a serious complication of both radiotherapy and immunotherapy, which could limit or even terminate the treatment of lung cancer. We found the incidence of pneumonitis to be higher than reported before. Four risk factors were associated with high-grade pneumonitis: PD-1 and PD-L1 inhibitors, SCC, and a higher ECOG score at diagnosis. Patients with more severe pneumonitis were more likely to have the progressive disease.

Further studies are needed to investigate and explain the suggested association between the pathogenesis of pneumonitis and poor response to lung cancer therapies. Similarly, the reported association between SCC and higher grade of pneumonitis (3 or above) is still not well-understood. Learning more about the risk factors of high-grade pneumonitis could improve treatment planning, clinical outcomes, and survival.

## Figures and Tables

**Figure 3:**
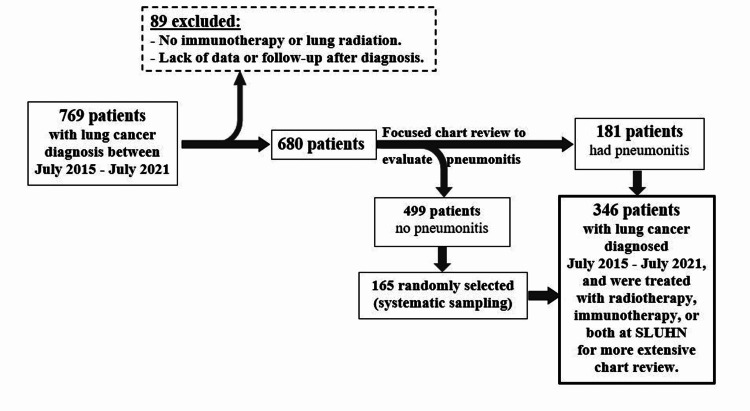
A flowchart of our retrospective study design SLUHN: St. Luke's University Health Network

## Appendices

**Table 10:** National Cancer Institute CTCAE pneumonitis grading system - version 5.0 (a) CTCAE: Common Terminology Criteria for Adverse Events. (b) ADL: activities of daily living; instrumental ADL refers to preparing meals, shopping for groceries or clothes, using the telephone, managing money, etc. (c) Self-care ADL refers to bathing, dressing, and undressing, feeding self, using the toilet, taking medications, and not bedridden Common Terminology Criteria for Adverse Events (CTCAE) Version 5.0 (2017) Accessed: April 22, 2022: https://ctep.cancer.gov/protocoldevelopment/electronic_applications/docs/CTCAE_v5_Quick_Reference_8.5x11.pdf

CTCAE^(a)^ Term	Grade 1	Grade 2	Grade 3	Grade 4	Grade 5
Pneumonitis	Asymptomatic	Symptomatic	Severe Symptoms	Life-Threatening	Death
A disorder characterized by inflammation focally or diffusely affecting the lung parenchyma.	clinical or diagnostic observations only; intervention not indicated	medical intervention indicated, limiting instrumental ADL^(b)^	Severe symptoms; limiting self-care ADL^(c)^; oxygen indicated	Life-threatening respiratory compromise; urgent intervention indicated (e.g., tracheostomy or intubation)	

**Table 11:** Revised Response Evaluation Criteria in Solid Tumors (RECIST) guideline version 1.1 (a) This includes the baseline sum if that is the smallest in the study. Adapted from Eisenhauer EA, Therasse P, Bogaerts J, et al.: New response evaluation criteria in solid tumors: revised RECIST guideline (version 1.1). Eur J Cancer. 2009, 45(2):228-247. 10.1016/j.ejca.2008.10.026

Response	Complete Response (CR)	Partial Response (PR)	Progressive Disease (PD)	Stable Disease (SD)	Not Evaluable (NE)
Definition	Disappearance of all target lesions. Any pathological lymph nodes (whether target or non-target) must have a reduction in the short axis to <10 mm.><10 mm.	≥ 30% decrease in the sum of diameters of target lesions, taking as reference the baseline sum diameters.	≥ 20% increase of ≥ 5mm in the sum of diameters of target lesions, taking as reference the smallest sum in the study^(a)^ The appearance of one or more new lesions is also considered progression.	Neither sufficient shrinkage to qualify for PR nor sufficient increase to qualify for PD, taking as reference the smallest sum diameters while in the study.	When no imaging/ measurement is done at all at a particular time point.

**Table 12:** Eastern Cooperative Oncology Group (ECOG) performance score. ECOG: Eastern Cooperative Oncology Group. Adapted from Oken MM, Creech RH, Tormey DC, et al.: Toxicity and response criteria of the Eastern Cooperative Oncology Group. American Journal of Clinical Oncology. 1982, 5(6): 649–655.

Grade	ECOG Performance Status
0	Fully active, able to carry on all pre-disease performance without restriction.
1	Restricted in physically strenuous activity but ambulatory and able to carry out work of a light or sedentary nature, e.g., light housework, office work.
2	Ambulatory and capable of all self-care but unable to carry out any work activities; up and about more than 50% of waking hours.
3	Capable of only limited self-care; confined to bed or chair > 50% of waking hours.
4	Completely disabled; cannot carry on any self-care; totally confined to bed or chair.
5	Dead.
